# Role of Intraoperative Near-Infrared Indocyanine Green Fluorescence Cholangiography in the Management of Acute Gangrenous Cholecystitis Secondary to Empyema of the Gallbladder in Mirizzi's Syndrome

**DOI:** 10.7759/cureus.68465

**Published:** 2024-09-02

**Authors:** Nikhil M Nagakumar, Sourav Panda, Vishal Lakhotia, Aditi Sachdeva, Rushil Jain

**Affiliations:** 1 General Surgery, Max Super Speciality Hospital, New Delhi, IND

**Keywords:** near-infrared fluorescence cholangiography, bile duct injury, indocyanine green, laparoscopic cholecystectomy, mirizzi syndrome, acute calculus cholecystitis

## Abstract

Mirizzi syndrome, although rare, is a potential complication of long-standing gallstone disease, particularly cholecystolithiasis. Due to the nonspecific nature of its symptoms, this condition often remains undiagnosed prior to surgery in most cases. While minimally invasive approaches are generally safe in expert hands, they can be challenging and entail the risk of bile duct injuries, often necessitating conversion to bail-out procedures. Delayed management of Mirizzi syndrome can lead to serious consequences, such as empyema of the gallbladder (GB), gangrene of the GB wall, perforation, and sepsis. Intraoperative indocyanine green fluorescence imaging during laparoscopic cholecystectomy can help delineate the biliary anatomy and prevent biliary tract injuries in difficult GBs like Mirizzi syndrome.

## Introduction

Mirizzi syndrome is one of the complications of chronic gallstone disease [1,2]. A stone lodged in Hartman’s pouch or the cystic duct, leading to external compression of the bile ducts, is suggested as the underlying mechanism [2]. Continuous pressure, persistent inflammation, ulceration, and subsequent infection can result in severe surgical emergencies, such as cholecysto-biliary or enteric fistulas, empyema of the gallbladder (GB), gangrene or perforation of the GB, and sepsis [3,4]. In these cases, inflammation and edema can hinder accurate anatomical assessment and identification of the common bile duct (CBD), leading to an increased risk of bile duct injuries [5,6]. Therefore, the use of intraoperative indocyanine green (ICG) near-infrared fluorescence (NIRF) cholangiography can help delineate biliary anatomy during laparoscopic cholecystectomy of difficult GBs (Mirizzi syndrome), thus reducing the risk of bile duct injuries.

## Case presentation

A 48-year-old male presented to the outpatient department (OPD) with complaints of pain in the right hypochondrium for seven days and yellowish discoloration of skin and sclera with high-colored urine for five days. The patient denied any history of fever or vomiting. The patient reported a one-year history of gallstone disease.

The patient had similar complaints six months back for which he was evaluated elsewhere and was diagnosed to have choledocholithiasis for which endoscopic retrograde cholangiopancreatography (ERCP) was done but the procedure failed to clear the stones from the CBD due to altered periampullary and biliary anatomy.

**Figure 1:**
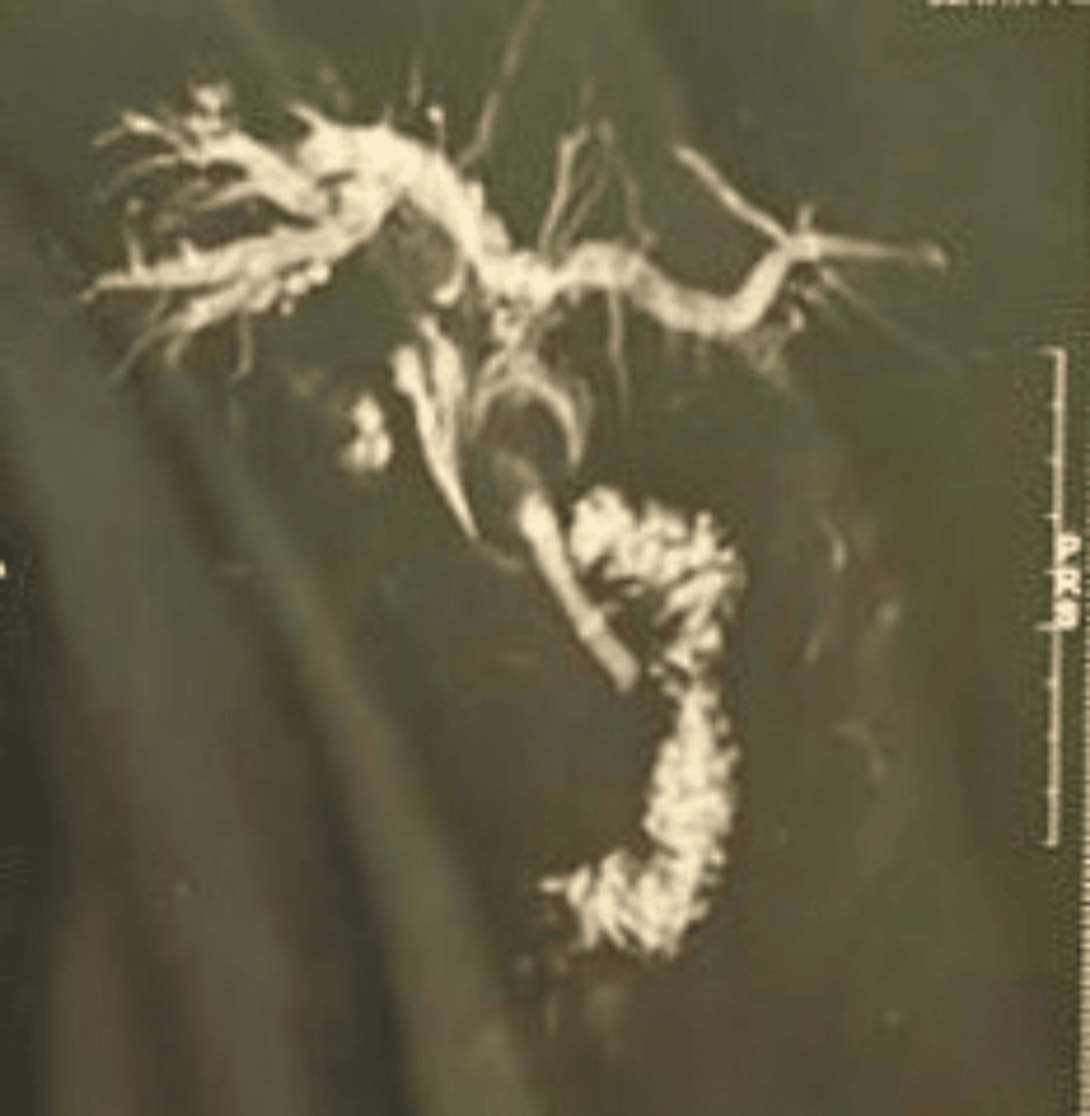
Magnetic resonance cholangiopancreatography (MRCP) Depicting MRCP findings of Type 1 Mirizzi syndrome

On physical examination in the OPD, the patient’s body temperature was 38.6 °C, pulse rate was 85 beats per minute, respiratory rate was 22 cycles per minute, and blood pressure was 110/72 mmHg. On palpation, there was a palpable mass in the right hypochondrium with associated tenderness. Murphy's sign was positive. Laboratory investigation results are tabulated in Table 1. The patient had raised INR for which preoperatively, the patient was started on intravenous vitamin K injections. USG abdomen revealed a pathological contracted GB with multiple calculi and an edematous wall with mild intrahepatic and extrahepatic biliary radical dilatation. Further magnetic resonance cholangiopancreatography (MRCP) was done to delineate biliary anatomy, which revealed a picture of acute calculous cholecystitis with irregularity and thinning of the GB wall probably suggestive of localized perforation, with a 2.5 cm calculus impacted at GB neck/cystic duct causing extrinsic compression over the common hepatic duct with mild dilatation of bilateral Intrahepatic biliary radicals, concluding it as a Type I Mirizzi syndrome (Figure 1). Csendes classification is used to classify Mirizzi syndrome into four types (Table 2) [4].

**Table 1:** Laboratory investigations ALP: Alkaline Phosphatase; GGT: Gamma Glutamyl Transpeptidase; INR: International Normalized Ratio.

Investigations	Results
Total leucocyte count	13,000/ mm^3^
Total serum bilirubin	5.2 mg/dL
Serum indirect bilirubin	2.61 mg/dL
Serum direct bilirubin	2.59 mg/dL
ALP	420 IU/L
GGT	156 IU/L
INR	1.6

**Table 2:** Csendes classification of Mirizzi syndrome

Csendes Type	Characteristics
Mirizzi type I	Extrinsic compression of the common hepatic duct is typically caused by stones lodged in the cystic duct or the infundibulum of the gallbladder.
Mirizzi type II	Presence of a cholecystobiliary fistula with a diameter that is one-third the circumference of the common hepatic duct wall.
Mirizzi type III	Presence of a cholecystobiliary fistula with a diameter exceeding two-thirds of the circumference of the common hepatic duct wall.
Mirizzi type IV	Presence of a cholecystobiliary fistula that involves the full circumference of the common hepatic duct wall.

**Figure 2:**
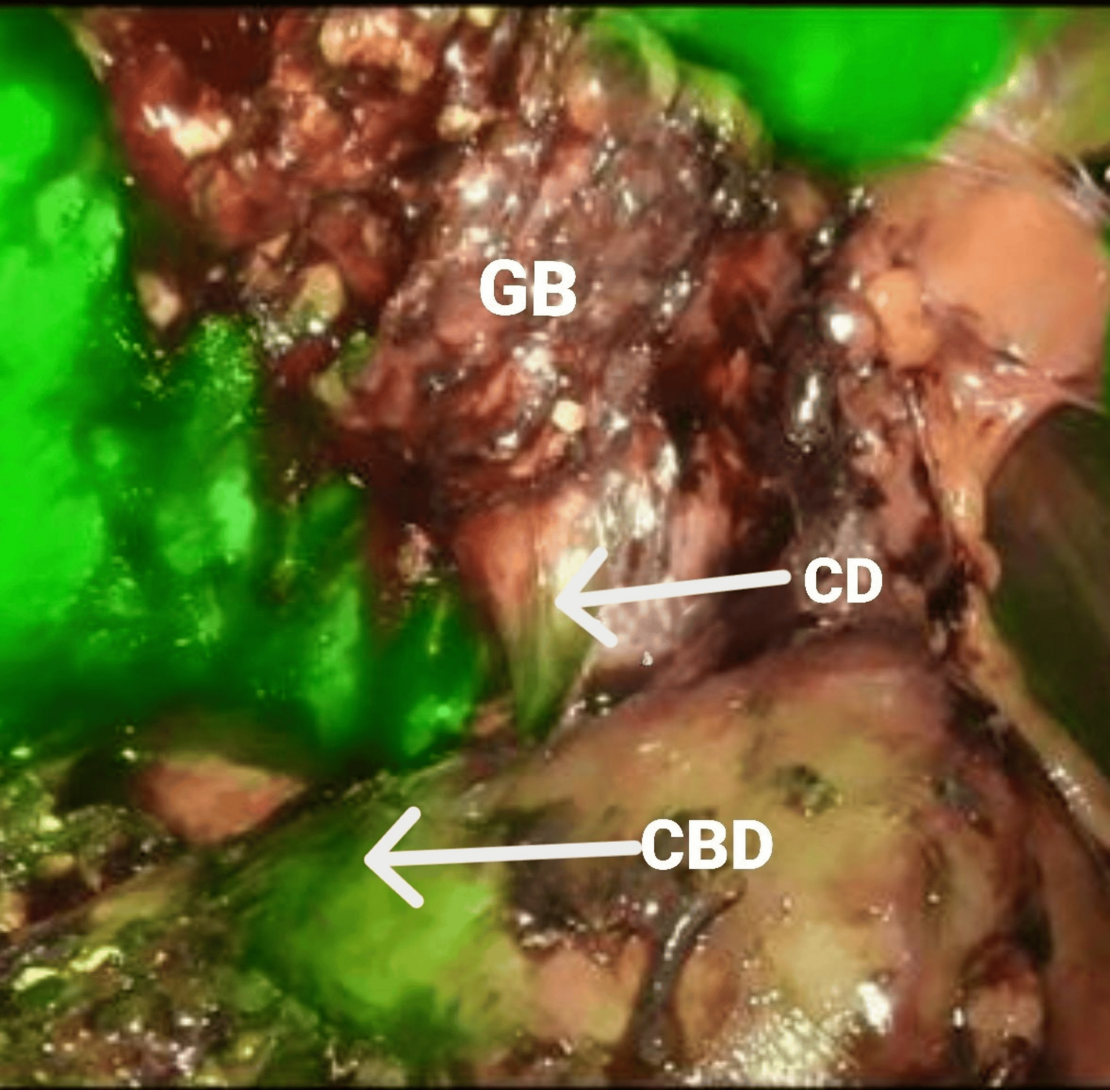
Intraoperative indocyanine green (ICG) near-infrared fluorescence (NIRF) cholangiography Intraoperative ICG NIRF cholangiography delineating biliary anatomy. CD: Cystic Duct; CBD: Common Bile Duct; GB: Gallbladder.

The patient was immediately scheduled for ERCP. Findings of ERCP revealed it to be a case of Type IV Mirizzi syndrome which was in contrary to the MRCP findings. Stones from the CBD were cleared and a stent was placed. The patient was then planned for diagnostic laparoscopy and proceed with laparoscopic cholecystectomy/subtotal cholecystectomy with hepaticojejunostomy if required intraoperatively. Intraoperative findings revealed a perforated emphysematous GB with a stone impacted at the cystic duct causing Type I Mirizzi syndrome. ICG NIRF cholangiography was done intraoperatively to delineate biliary anatomy (Figure 2) which played a major role in preventing injury to bile ducts and conversion to open cholecystectomy.

Technique of ICG NIRF cholangiography

A vial of ICG (25 mg) was diluted with 10 mL of sterile water. Once reconstituted, the ICG solution was injected via the intravenous route using a dosage of 0.35 mg/Kg before induction of anesthesia. The ICG-NIRF cholangiography was activated by pushing a button on the camera head and allowed real-time fluorescent visualization of extrahepatic biliary structures before dissection of the Calot’s triangle. 

**Figure 3:**
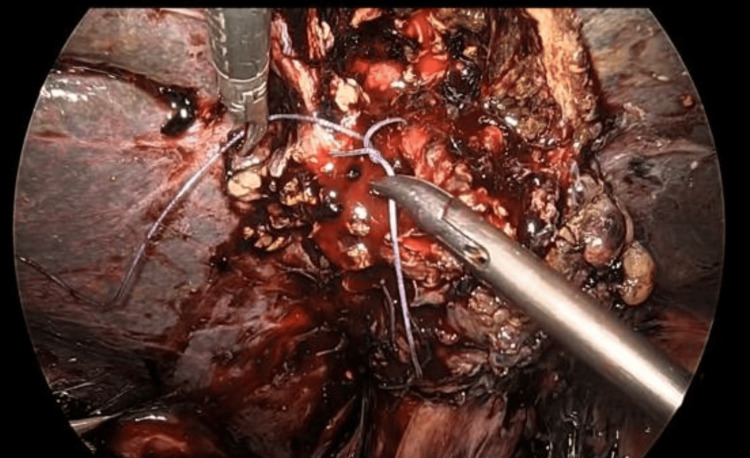
Suturing the flap of the posterior gallbladder wall with a cystic stump

The GB was removed partially, a healthy flap raised from the sloughed out posterior wall of the GB and was sutured to the cystic duct stump with 2-0 Vicryl (Figure 3). Subhepatic drain was placed and surgery was uneventful. The patient recovered well and was discharged in stable condition on postoperative day 4. The patient was comfortable on further follow up.

## Discussion

Mirizzi syndrome, although rare, is a potential complication of long-standing gallstone disease, particularly cholecystolithiasis [1,2]. A stone lodged in the cystic duct or Hartman’s pouch, leading to external compression of the bile ducts, is the fundamental cause [2]. Continuous pressure, persistent inflammation, ulceration, and subsequent infection can result in severe surgical complications, such as cholecysto-biliary or enteric fistulas, empyema of the GB, gangrene or perforation of the GB, and sepsis. [3,4].

According to the Tokyo Guidelines 2018 (TG18), laparoscopic cholecystectomy is recognized as a safe and effective surgical treatment for acute calculous cholecystitis [7,8]. However, accurate identification of the biliary anatomy during surgery is crucial to avoid iatrogenic injury to the bile ducts. Inflammation and edema in these cases can obstruct proper anatomical delineation and identification of the CBD, leading to a higher risk of bile duct injuries [5,6]. Iatrogenic injury to the bile duct is a known complication of laparoscopic cholecystectomy (around 0.5% to 6% in elective surgeries) and is mainly due to the incorrect identification of the biliary anatomy during surgery [9].

NIRF cholangiography with ICG has emerged as an innovative method for intraoperative identification and mapping of the extrahepatic biliary system. The use of ICG-assisted laparoscopic cholecystectomy in elective cases of acute calculous cholecystitis is safe and effective [10].

We present a rare case of acute gangrenous cholecystitis secondary to empyema of the GB in Mirizzi's syndrome, where the patient was planned for early laparoscopic cholecystectomy after ERCP as MRCP suggested localized GB perforation. Further delays could have resulted in biliary peritonitis and sepsis. Although there is controversy surrounding the use of laparoscopic procedures for this condition, we opted for a laparoscopic approach, aspirated the empyema, opened the GB wall, extracted the stone, and performed a subtotal cholecystectomy. NIRF cholangiography using ICG helped us delineate the biliary anatomy and prevent injury to biliary ducts and also conversion to open surgery.

## Conclusions

Performing a laparoscopic cholecystectomy in cases of Mirizzi syndrome is highly challenging due to chronic inflammation, edema, and adhesions, which frequently result in bile duct injuries. NIRF cholangiography using ICG is a novel technique, which can be used intraoperatively to delineate biliary anatomy in difficult GBs like Mirizzi syndrome to prevent injury to the biliary ducts.
